# Modified vaccinia Ankara vaccine expressing Marburg virus-like particles protects guinea pigs from lethal Marburg virus infection

**DOI:** 10.1038/s41541-020-00226-y

**Published:** 2020-09-02

**Authors:** Delphine C. Malherbe, Arban Domi, Mary J. Hauser, Michelle Meyer, Bronwyn M. Gunn, Galit Alter, Alexander Bukreyev, Farshad Guirakhoo

**Affiliations:** 1 Department of Pathology, University of Texas Medical Branch, Galveston, TX USA; 2 Galveston National Laboratory, Galveston, TX USA; 3 GeoVax Inc., Atlanta, GA USA; 4 Ragon Institute of MGH, MIT, and Harvard, Cambridge, MA USA; 5 Department of Microbiology & Immunology, University of Texas Medical Branch, Galveston, TX USA

**Keywords:** Biologics, Vaccines

## Abstract

We introduce a new vaccine platform against Marburg virus (MARV) combining the advantages of the immunogenicity of a highly attenuated vaccine vector (Modified Vaccinia Ankara, MVA) with the authentic conformation of virus-like particles (VLPs). Our vaccine, MVA–MARV–VLP, expresses the minimal components of MARV VLPs: the envelope glycoprotein GP and the matrix protein VP40. Electron microscopy confirmed self-assembly and budding of VLPs from infected cells. Prime/boost vaccination of guinea pigs with MVA–MARV–VLP-elicited MARV-specific binding and neutralizing antibody responses. Vaccination also induced Fc-mediated innate immune effector functions including activation of NK cells and antibody-dependent phagocytosis by neutrophils and monocytes. Inoculation of vaccinated animals with guinea pig-adapted MARV demonstrated 100% protection against death and disease with no viremia. Therefore, our vaccine platform, expressing two antigens resulting in assembly of VLPs in the native conformation in vaccinated hosts, can be used as a potent vaccine against MARV.

## Introduction

**Fig. 1:**
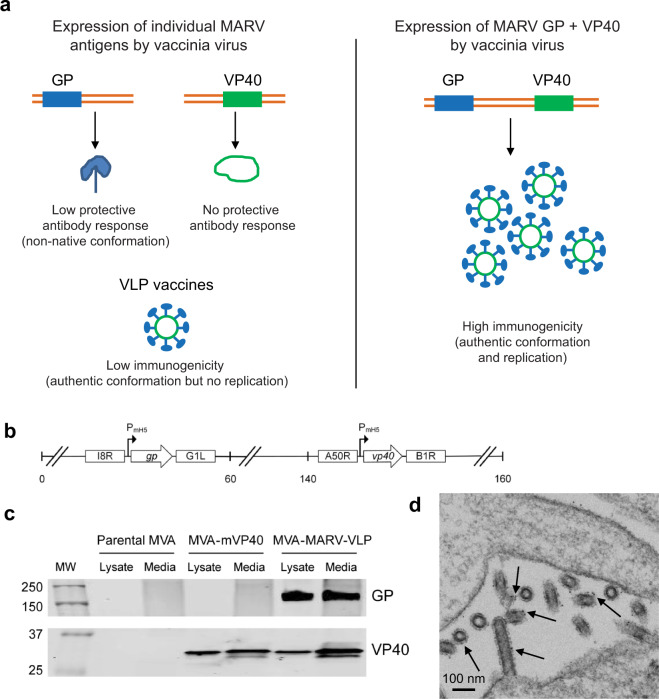
Vector design and VLP expression. **a** Advantages of the MVA-based VLP vaccine platform compared with MVA vector-based vaccines and VLP vaccines. **b** Vector Map. MARV *gp* gene was inserted between the I8R and G1L sequences of MVA, and *vp40* was inserted into the restructured and modified deletion III. These insertion sites have been identified as supporting high expression and insert stability. Positions are given in kilobase pairs (kbp) in the MVA genome. **c** Western blot for MARV GP and VP40 expression. DF1 cells infected with a MOI of 0.5 FFU/cell of parental MVA, MVA–mVP40, or MVA–MARV–VLP. Supernatants and cell lysates were run on a 4–12% SDS-PAGE before transfer and detection with antibodies specific for MARV GP and MARV VP40, respectively. The two fragments shown are parts of the same gel. **d** Electron microscopic analysis of expression of virus-like particles from MVA–MARV–VLP. HEK293 cells were infected with MVA–MARV for 24 h, stained with a human anti-GP antibody, fixed with 1% glutaraldehyde in 0.1 M phosphate buffer and incubated in 50 mM glycine to block residual aldehyde. Following incubation in goat anti-human secondary antibody conjugated to ultra-small gold particles, silver enhancement was performed to increase the size of gold particles for subsequent viewing on a JEOL JEM-1400. Localization of some gold beads is indicated by black arrows.

Marburg virus (MARV) is a member of the *Filoviridae* family which causes a severe human disease with a 24–88% case fatality rate^1^. There are currently no licensed vaccines or therapeutics against the disease caused by MARV but several vaccine platforms have been advanced including vesicular stomatitis virus^2,3^, adenovirus^4^, and DNA vectors^5^. The highly attenuated live vector modified vaccinia Ankara (MVA)-based vaccine expressing only the single glycoprotein (GP) protein of Ebola virus, another filovirus, was not protective in vivo^6^ possibly due to non-authentic GP conformation (Fig. 1a). Another approach is to generate virus-like particles (VLPs)^7,8^ in vitro to be used as subunit protein vaccines. VLPs mimic the native conformation of viral particles (Fig. 1a) and can be very immunogenic, but since they do not replicate in the host, they require multiple doses for complete protection. In addition, these vaccines may need to be administered with adjuvants and are expensive to manufacture. Here we designed, constructed, and tested a novel live vaccine MVA–MARV–VLP, which is based on MVA that expresses the minimal components of MARV to generate VLPs: the envelope glycoprotein GP and the matrix protein VP40. As such, the MVA–MARV–VLP vaccine platform combines the advantages of the authentic conformation of VLPs with the immunogenicity of a replicating but a highly attenuated MVA vaccine vector (Fig. 1a). Testing of MVA–MARV–VLP demonstrated induction of various levels of MARV-neutralizing antibodies and Fc-mediated antibody protective effects; all vaccinated animals were protected from death and disease against lethal MARV infection.

## Results

### Design and development of MVA–MARV–VLP

To generate the vaccine candidate, GP and VP40 cDNA sequences of MARV strain Musoke, were selected, which were previously used for experimental vaccines against MARV^2,9,10^. To avoid instability, the cDNA sequences were codon optimized for the MVA vaccinia virus genome as described in Materials and Methods and inserted between MVA essential genes. Specifically, GP was inserted between the I8R and G1L genes, and VP40 was inserted into the restructured and modified deletion III between the A50R and B1R genes (Fig. 1b). MARV GP and VP40 expression in continuous chicken fibroblast DF1 cells infected with the vaccine construct was confirmed by western blot analysis (Figs. 1c, S2). Self-assembly of MARV VLPs in human HEK293 cells infected with MVA–MARV–VLP for 24 h was confirmed by thin section transmission electron microscopy which involved staining of GP with small gold particles (Fig. 1d). Thus, MVA–MARV–VLP-infected cells produce budding MARV VLPs.

### Antibody responses elicited by MVA–MARV–VLP vaccine

The immunogenicity and efficacy of the MVA–MARV–VLP vaccine was evaluated in the guinea pig animal model. Dunkin–Hartley guinea pigs (five animals per group) were vaccinated twice intramuscularly with 10^8^ 50% tissue culture infectious doses (TCID_50_) per animal of MVA–MARV–VLP on days 0 and 29, while the mock-vaccinated control group received saline solution. At day 56, guinea pigs were inoculated intraperitoneally with a lethal dose of 10^3^ plaque forming units (PFU) guinea pig-adapted MARV strain Angola (heterologous strain), the most virulent strain of MARV^11^, which caused the most deadly of known outbreaks of the virus with 90% lethality^12^.

**Fig. 2:**
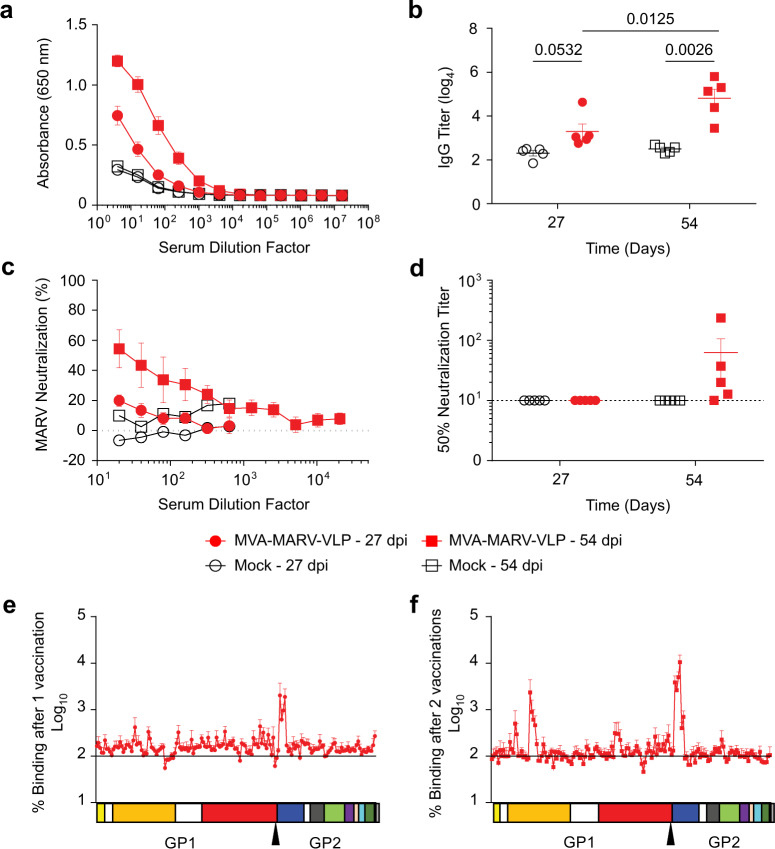
MVA–MARV–VLP vaccine elicits binding and neutralizing antibodies. Guinea pigs at five animals per group were vaccinated or mock-vaccinated at days 0 and 29. Sera collected 27 days post inoculation were assessed for their ability to bind to MARV Angola GP (**a**, **b**) and to neutralize MARV strain Angola (**c**, **d**). The MVA–MARV–VLP group is shown in red, the control group in black. *P* values were determined by 2-way ANOVA followed by Bonferroni post-test. Sera collected after the first (**e**) and the second (**f**) vaccine dose were analyzed for binding to peptides matching the protein sequence of MARV Angola GP. Values are Log_10_ mean ± SEM representing the binding of sera from three responders as a percentage of the binding of pre-immune sera. A linear map of MARV GP domains is displayed as a diagram below each graph: signal peptide (yellow, 1–18 aa); receptor-binding domain (orange, 38–188 aa); mucin-like domain (red, 257–501 aa); wing region (dark blue, 436–501 aa); internal fusion loop (dark gray, 511–552 aa); N-terminal heptad repeat (light green, 553–596 aa); CX6CC motif (purple, 602–610 aa); C-terminal heptad repeat (salmon, 616–633 aa); membrane-proximal external region (cyan, 634–655 aa); transmembrane anchor (dark green, 659–667 aa); and cytoplasmic tail (light gray, 674–681 aa). The furin cleavage site is marked by the triangle.

**Fig. 3:**
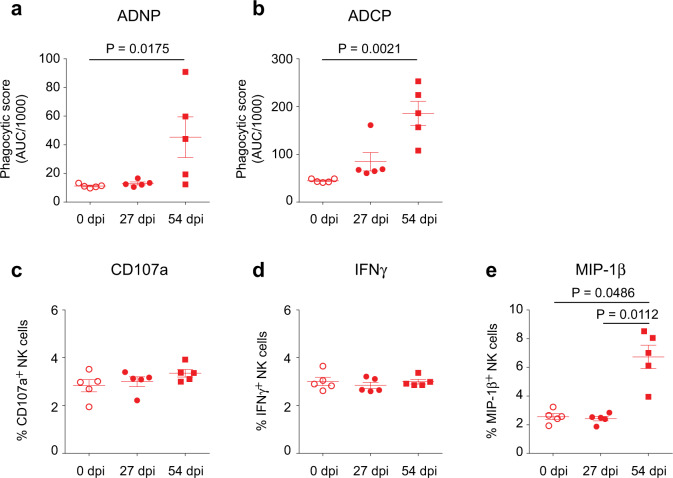
MVA–MARV–VLP vaccine elicits antibodies capable of inducing several Fc-dependent innate immune effector functions. Guinea pig sera from the MVA–MARV–VLP vaccine group were incubated with GP coated fluorescent beads which were incubated with either human neutrophils (**a**) or THP-1 monocytes (**b**). Cells were then analyzed by flow cytometry to quantify phagocytic intake of fluorescent bead/antibody complexes. Guinea pig sera were incubated on plates coated with MARV GP. Freshly isolated human NK cells were added to the plates and assessed by flow cytometry for expression of three activation markers: CD107a (**c**), IFNγ (**d**), and MIP-1β (**e**). *N* = 5 animals per group. *P* values were determined by Kruskal–Wallis analysis followed by Dunn’s multiple comparison test.

Because the strain used for inoculation is not homologous to our MARV Musoke-based vaccine, we monitored the development of MARV Angola, rather than Musoke, strain-specific humoral responses as they would be more predictive of protection from infection. Thus, serum samples collected on days 27 and 54 were assayed to determine MARV-specific antibody responses after the first and second vaccinations (Figs. 2, 3). In the vaccinated group, MARV GP-specific IgG binding antibody titers were detectable, but relatively low after the first vaccine dose (Fig. 2a) and were significantly increased by the second dose (Fig. 2b). A peptide array identified two main MARV linear epitopes targeted by MVA–MARV–VLP-induced antibodies (Fig. 2e, f): the receptor-binding site (RBS) (residues 61–71 and 97–115) and the wing region (residues 445–471). Neutralizing antibody titers against MARV strain Angola were not detectable after the first dose, but detectable in most animals upon boosting (Fig. 2c); the mean values did not reach significance due to the lack of neutralizing activity in one animal and very low activity in another animal (Fig. 2d).

### Induction of Fc-mediated protective effects

While a neutralizing antibody response is vitally important for the efficacy of most vaccines, antibody Fc domain-mediated responses can also play an important role in protection. In our assays, human cells were used for guinea pig cells since it was shown previously that guinea pig antibodies can activate human immune cells^13^. Vaccination with MVA–MARV–VLP-elicited MARV Angola strain-specific Fc-mediated innate immune effector functions including phagocytosis by neutrophils (ADNP, Figs. 3a, S1) and monocytes (ADCP, Figs. 3b, S1); both effects were readily detectable in most animals after the second vaccine dose. The MVA–MARV–VLP vaccine also partially activated NK cells; interestingly, the second vaccine dose induced upregulation of MIP-1β (Figs. 3e, S1) but not CD107a or interferon-γ (Figs. 3c, d, S1). Notably, while activation of NK cells facilitates antibody-mediated protection against EBOV, it is not absolutely required^14^.

### Protection from MARV infection by MVA–MARV–VLP vaccine

**Fig. 4:**
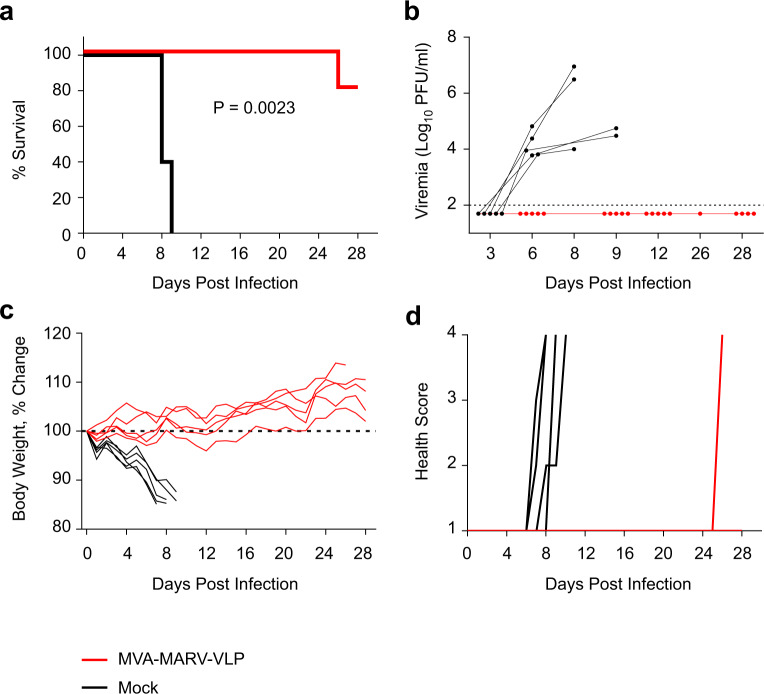
MVA–MARV–VLP vaccine fully protects vaccinated guinea pigs from MARV lethal challenge. Guinea pigs were vaccinated at days 0 and 29 and challenged with guinea pig-adapted MARV strain Angola at day 56. Survival (**a**), viremia (**b**), % change in individual body weight (**c**), and individual disease scores (**d**). In panel **b**, for the samples in which no virus was detected, the values twofold below the limit of detection (indicated by the dotted line) were assigned. MVA–MARV group is shown red, mock-vaccinated control group in black. Five animals per group. Survival *P* value was determined by Log Rank (Mantel Cox) test.

Four weeks after the second vaccination, guinea pigs were exposed to 1000 PFU of guinea pig-adapted MARV strain Angola^5^ by the intraperitoneal route (Fig. 4a). In the control group, a significant loss of weight (*P* < 0.0001) was observed in all animals starting on day 3 (Fig. 4c), followed by other signs of infection starting on day 7 (Fig. 4d). Viremia was detected in all control animals starting on day 6 (Fig. 4b). On day 8 or 9, all animals reached moribund condition and were euthanized (Fig. 4a); a maximum level of viremia was detected upon euthanasia. In contrast, guinea pigs vaccinated with MVA–MARV–VLP demonstrated no loss of weight (Fig. 4c) or other signs of the disease (Fig. 4d) and no detectable viremia (Fig. 4b) at any time point tested. One animal in the vaccine group was euthanized on day 26 (Fig. 4a) for reasons unrelated to MARV infection as no viremia was detected (Fig. 4b); in addition, no MARV was detected in liver, spleen, kidney, lung, and brain collected at necropsy (Fig. S3). Thus, the MVA–MARV–VLP-vaccinated guinea pigs remained aviremic during the entire observation period, indicative of a strong immunity against MARV infection.

## Discussion

This study presents the development and testing of a novel vaccine platform against MARV. While VLP-based protein vaccines present antigens in a native conformation and are highly immunogenic, they are expensive to produce, may require potent adjuvants, stabilization against aggregations, extensive purifications, and two to three doses to confer protection from death but not from disease^8,15^. On the other hand, vaccines based on chimeric viral vectors in which the vector protein is replaced with filovirus GP, represent a new virus with a new tropism, may shed to the environment from vaccinated subjects and may be reactogenic^16^. Furthermore, the MVA virus vector does not effectively replicate in mammalian cells and therefore is safe, but generally requires multiple injections or priming with a heterologous vector such as adenovirus-based vector^17^. Our new MVA–MARV–VLP vaccine platform is based on the MVA vector and expresses the minimal MARV genes GP and VP40 to produce self-assembling VLPs in vivo. Thus, this novel vaccine platform combines the advantages of the safety of the MVA vector with the immunogenicity of the noninfectious VLPs as MARV antigens are displayed in authentic conformation.

Testing protective efficacy of MVA–MARV–VLP against heterologous MARV infection in guinea pigs demonstrated induction of a strong protective immune response. The first vaccine dose elicited GP-binding antibodies which increased upon boosting. Following two doses, MARV-neutralizing antibodies were also detected in most animals. The second vaccine dose induced antibodies that targeted GP epitopes associated with protection. Specifically, the identified epitope in RBS overlaps with the epitope of MR78, an antibody targeting residues essential for viral entry^18,19^, while the wing region epitope overlaps with the epitope of mouse antibodies 30G3, 30G4, 54G2, and 30G5^20^ and antibodies MR228 and MR235 from a human survivor^21^. Antibodies 30G5^20^ and MR228^21^ are fully protective from death in mice, while MR78 is fully protective from death in guinea pigs^22^. In addition, the second dose induced Fc-mediated protective effects: phagocytosis by neutrophils and monocytes and activation of NK cells. A recent study demonstrated protection against MARV by a non-neutralizing monoclonal antibody MR228^21^. Since two animals demonstrated little to no MARV-neutralizing antibodies, but were fully protected against the infection, both antibody neutralizing activity and Fc-mediated immune functions may be responsible for protection. Contribution of T cells in protection also cannot be ruled out.

The infection experiment was performed with the heterologous MARV strain Angola. The Angola strain was responsible for the largest outbreak ever documented for MARV, which resulted in 227 deaths out of 252 total cases (a 90% mortality rate) in Uige, Angola^23^. Moreover, the Angola strain was also shown to be more virulent in nonhuman primates than the Musoke strain^24^. GP from the Angola strain differs from that of the Musoke strain by 7.20%^23^; thus, the study we performed represents the most stringent efficacy test for a MARV vaccine. In conclusion, our novel vaccine platform delivering antigens in a native conformation as self-assembling VLPs by an improved replication-deficient vector was used to develop a potent vaccine against MARV. This study, along with the recently demonstrated applicability of the platform to Ebola^25,26^ and Lassa^27^ viruses, demonstrate the versatility of the vaccine platform and its potential use for additional emerging viral pathogens, such as SARS-CoV-2.

## Methods

### Generation and characterization of MVA–MARV–VLP vaccine

The MVA–MARV–VLP vaccine construction was performed as previously described^26^ using MARV strain Musoke GP and VP40 sequences (GenBank Accession number JX458835.1). Briefly, MARV genes were codon optimized for vaccinia virus genome to avoid instability of the construct. Codon optimizations included introduction of silent mutations to interrupt homo-polymer sequences (>4G/C and >4A/T) to reduce RNA polymerase errors that could lead to frameshifts and to remove vaccinia-specific terminators that could lead to premature termination^28^. Genes were inserted in a parental MVA that had been harvested in 1974 before the appearance of bovine spongiform encephalopathy (BSE) and plaque purified three times using certified reagents from sources free of BSE. The GP sequence was inserted between two essential vaccinia genes (I8R and G1L) using the pLW73 shuttle vector, and the VP40 sequence was placed in a modified and restructured insertion site III using the pLW76 shuttle vector (Wyatt and Moss, unpublished data). MVA–MARV VLPs were produced in DF1 cells as described. To confirm expression of GP and VP40, DF1 cells infected with MVA–MARV–VLP or the parental MVA (empty vector) at a MOI of 0.5 FFU/cell for 48 h at 37 °C. Subsequently, cells were lysed and the proteins were separated by 4–12% SDS-PAGE under denaturing and reducing conditions. Proteins were transferred to nitrocellulose, and the membrane was stained with anti-MARV GP (IBT cat # 0303-007) and anti-MARV VP40 (IBT cat # 0303-001) antibodies and visualized with the Li-Cor Odyssey imaging system. To confirm the formation of VLPs by electron microscopy, HEK293 cells were infected with MVA–MARV–VLP for 24 h, stained with a rabbit anti-GP antibody, fixed with 1% glutaraldehyde in 0.1 M phosphate buffer and incubated in 50 mM glycine to block residual aldehyde. Following incubation in goat anti-human secondary antibody conjugated to 6 nm gold particles (Aurion, Netherlands), silver enhancement was done to increase the size of gold particles for subsequent viewing on a JEOL JEM-1400 electron microscope.

### Vaccination and MARV infection

This study was carried out in strict accordance with the recommendations described in the Guide for the Care and Use of Laboratory Animals of the National Research Council and the United States Department of Agriculture. UTMB is an AAALAC-accredited institution and all animal work was approved by the IACUC Committee of UTMB. All efforts were made to minimize animal suffering and all procedures involving potential pain were performed with the appropriate anesthetic or analgesic. The number of animals used in this study was scientifically justified based on statistical analyses of virological and immunological outcomes. Nine-week-old Dunkin–Hartley female guinea pigs (Charles Rivers Laboratories) were anesthetized with 5% isoflurane for all procedures. On days 0 and 29, the MVA–MARV–VLP vaccine group (five animals) was inoculated with 10^8^ TCID_50_ per animal in 100 µl injection volume via the intramuscular route, while the control group (*N* = 5 animals) received 100 µl saline solution via the intramuscular route. Retro-orbital blood collections were performed on days −1 (1 day prior the first immunization), 27 and 54. On day 56 vaccinated and control animals were exposed intraperitoneally to the targeted dose of 10^3^ PFU of guinea pig-adapted MARV strain Angola. Animals were monitored up to three times per day for weight loss and signs of disease and were euthanized when they reached the moribund state. Remaining animals were euthanized 28 days post infection. Retro-orbital blood collections were performed from surviving animals 3, 6, 9, 12, and 28 days post virus inoculation. Viremia was determined by plaque assay as previously described^13^. Briefly, MARV plaques were immunostained with 1 µg/ml human mAb cocktail (MR78, MR186, MR235, a gift from Dr. Crowe, Vanderbilt University Medical Center) and horse radish peroxidase-conjugated goat anti-human IgG at dilution 1:2,000 (SeraCare, #5220-0456).

### Analysis of the antibody response

Serum samples were tested for their ability to bind MARV strain Angola GP (IBT Bioservices, #0506-015) by ELISA as previously described^13^. Serum samples were also tested for neutralization of MARV strain Angola by plaque reduction assay on Vero E6 cell monolayers as previously described^13^. Serum samples were tested for their ability to bind overlapping 15-mer peptides covering the length of MARV strain Angola GP in a peptide microarray (JPT Peptide Technologies, Berlin, Germany) as previously described^13^.

### Isolation of neutrophils

Peripheral whole blood from human donors was collected into an acid citrate dextrose Vacutainer (BD Biosciences). Blood was transferred to a 50 ml conical tube and diluted 1:10 with ammonium chloride potassium lysis buffer (ThermoFisher Scientific) to lyse red blood cells. Following a gentle inversion to mix, the blood and lysis buffer were incubated together for 5 min at the room temperature. White blood cells were collected by centrifugation at 500 × *g* for 5 min. Cell pellet was resuspended and washed twice in cold phosphate buffered saline (PBS) and collected by centrifugation at 500 × *g* for 5 min followed by a third wash in complete growth medium (RPMI1640 containing 10% FCS, 2mM l-glutamine, 1% penicillin/streptomycin, and 1% HEPES). White blood cells were counted and resuspended to a final concentration of 5 × 10^5^ cells/ml in complete growth medium for immediate use in the phagocytosis assay. To identify phagocytosis by the neutrophils, the white blood cells were stained with anti-CD66b pacific blue (clone G10F5; BioLegend), anti-CD3 AlexaFluor 700 (clone UCHT1; BD Biosciences), and anti-CD14 APC-Cy7 (clone MφP9; BD Biosciences). Neutrophils were defined as positive for a high side scatter area (SSC-A^high^), CD66b^+^, CD3^−^, and CD14^−^.

### Isolation of natural killer cells

Primary human NK cells were enriched from a buffy coat by negative selection with RosetteSep (StemCell). The buffy coat was transferred to a 50 ml conical tube and the total volume was measured. RosetteSep Human NK cell Enrichment cocktail (StemCell Technologies) was added at 50 µl per ml of buffy coat and incubated at the room temperature for 20 min. Following incubation, the buffy coat was diluted 1:1 with 1X sterile PBS. NK cells were isolated using density centrifugation by overlaying the buffy coat over Histopaque 1077 (Millipore Sigma) in a SepMate tube (StemCell) and centrifuging at 1200 × *g* for 10 min. Supernatant was decanted into a new 50 ml conical tube, and cells were pelleted at 500 × *g* for 10 min. Cell pellet was resuspended and washed with sterile PBS followed by a second wash in complete growth medium (RPMI1640 containing 10% FCS, 2mM l-glutamine, 1% penicillin/streptomycin, and 1% HEPES). NK cells were resuspended to a final concentration of 1.5 × 10^6^ cells/ml in complete growth medium supplemented with 1 ng/ml of recombinant human IL-15 (StemCell Technologies). Cells were incubated at 37 °C 5% CO_2_ overnight until ready for use in the assay. Prior to use in the assay, cells were recounted, and volume adjusted to needed concentration of cells in complete growth medium.

### Analysis of Fc-mediated protective effects

To analyze antibody-mediated phagocytosis, recombinant GP of MARV strain Angola was biotinylated and coupled to 1 µm FITC^+^ Neutravidin beads (Life Technologies). White blood cells were isolated from donor peripheral blood by lysis of red blood cells, followed by three washes with PBS. Phagocytosis by human neutrophils and by human THP-1 monocytes was assessed as previously described^13^. To analyze antibody-dependent NK cell activation, recombinant GP of MARV strain Angola lacking the transmembrane domain (IBT Bioservices, #0506-015) and freshly isolated human NK cells were used for experiments to assess induction of innate immune effector functions as previously described^13^.

### Biocontainment work

Work with MARV was performed in the BSL-4 facilities of the Galveston National Laboratory. Staff had the appropriate training and U.S. government permissions and registrations for work with MARV.

### Statistical analyses

2-way ANOVA followed by Bonferroni post-test; Kruskal–Wallis analysis followed by Dunn’s multiple comparison test and Log Rank (Mantel Cox) test were performed with GraphPad Prism for Windows (version 6.07). *P* < 0.05 was considered significant.

### Reporting summary

Further information on research design is available in the Nature Research Reporting Summary linked to this article.

## Supplementary information

Supplementary Information

Reporting Summary

## Data Availability

The datasets generated during and/or analyzed during the current study are available from the corresponding author on reasonable request.
