# Supplementary material for: Modified vaccinia Ankara vaccine expressing Marburg virus-like particles protects guinea pigs from lethal Marburg virus infection
Source: NPJ Vaccines. 2020 Sep 2;5:78. doi: 10.1038/s41541-020-00226-y (PMC7468113; doi:10.1038/s41541-020-00226-y)
Supplement: Supplementary file 1 — Supplementary Information [file 41541_2020_226_MOESM1_ESM.pdf]

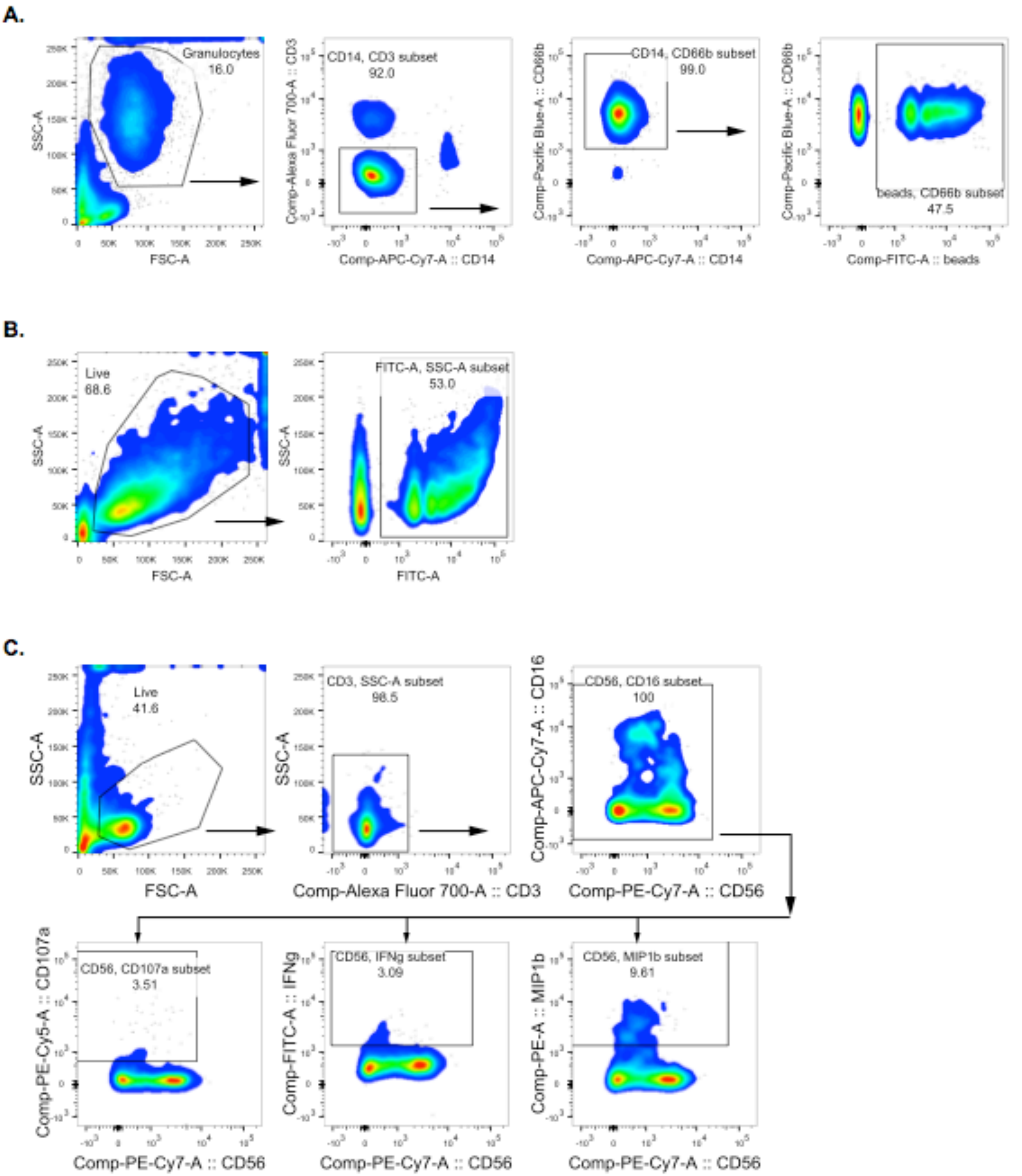


**Figure S1. Representative flow cytometry plots and gating strategy.** A: ADNP, B: ADCP, C: activation of NK cells.

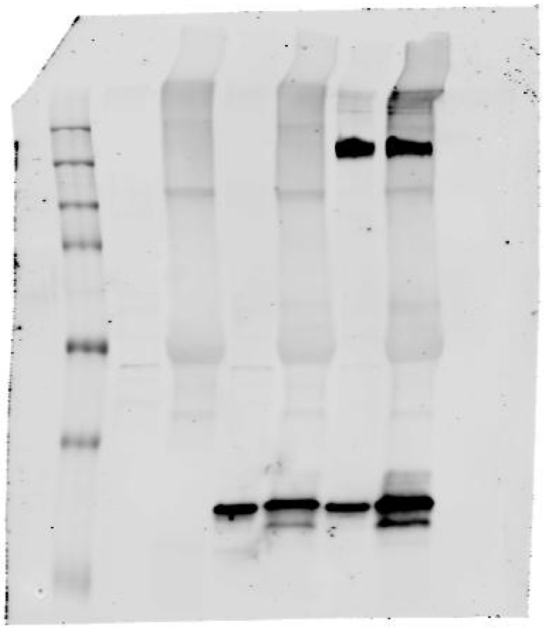

**Figure S2. Full image of Western blot  shown in Figure 1C.**

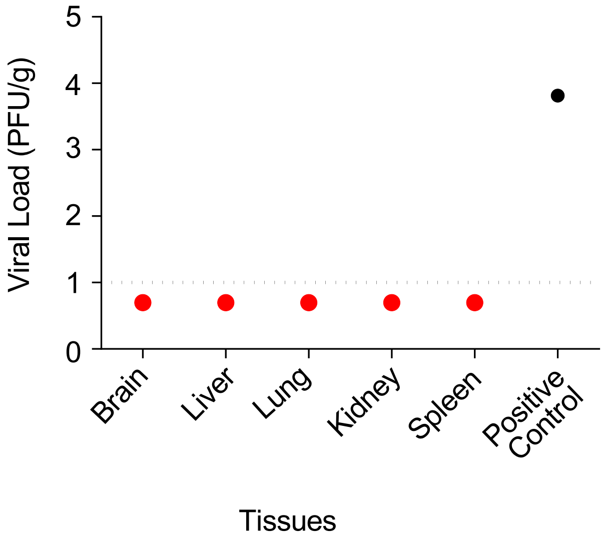


**Figure S3. MARV tissue viral loads in the vaccinated animal euthanized on day 26 after MARV inoculation.** Tissues (brain, liver, lung, kidney and spleen) from the vaccinated animal euthanized on day 26 after inoculation with MARV were harvested at necropsy and and homogenized in minimal essential medium. Clarified tissue homogenates were analyzed for MARV by plaque assay. As no virus was detected, values two-fold below the level of detection (indicated by the dotted line) were assigned. The viral titers are expressed in PFU per gram of tissue. In parallel, a blood sample from a non-vaccinated animal was analyzed for MARV as a positive control for virus detection; the virus titer for this sample is expressed in PFU/ml.
