# Supplementary material for: Modified vaccinia Ankara vaccine expressing Marburg virus-like particles protects guinea pigs from lethal Marburg virus infection
Source: NPJ Vaccines. 2020 Sep 2;5:78. doi: 10.1038/s41541-020-00226-y (PMC7468113; doi:10.1038/s41541-020-00226-y)
Supplement: Supplementary file 2 — Reporting Summary [file 41541_2020_226_MOESM2_ESM.pdf]

natureresearch

Corresponding author(s): Alexander Bukreyev, PhD

# Reporting Summary

Nature Research wishes to improve the reproducibility of the work that we publish. This form provides structure for consistency and transparency in reporting. For further information on Nature Research policies, see Authors & Referees and the Editorial Policy Checklist.

## Statistical parameters

When statistical analyses are reported, confirm that the following items are present in the relevant location (e.g. figure legend, table legend, main text, or Methods section).

| n/a | Confirmed | |
|---|---|---|
| ☐ | ☒ | The exact sample size (*n*) for each experimental group/condition, given as a discrete number and unit of measurement |
| ☐ | ☒ | An indication of whether measurements were taken from distinct samples or whether the same sample was measured repeatedly |
| ☐ | ☒ | The statistical test(s) used AND whether they are one- or two-sided<br>*Only common tests should be described solely by name; describe more complex techniques in the Methods section.* |
| ☐ | ☒ | A description of all covariates tested |
| ☐ | ☒ | A description of any assumptions or corrections, such as tests of normality and adjustment for multiple comparisons |
| ☐ | ☒ | A full description of the statistics including central tendency (e.g. means) or other basic estimates (e.g. regression coefficient) AND variation (e.g. standard deviation) or associated estimates of uncertainty (e.g. confidence intervals) |
| ☐ | ☒ | For null hypothesis testing, the test statistic (e.g. *F*, *t*, *r*) with confidence intervals, effect sizes, degrees of freedom and *P* value noted<br>*Give P values as exact values whenever suitable.* |
| ☒ | ☐ | For Bayesian analysis, information on the choice of priors and Markov chain Monte Carlo settings |
| ☒ | ☐ | For hierarchical and complex designs, identification of the appropriate level for tests and full reporting of outcomes |
| ☒ | ☐ | Estimates of effect sizes (e.g. Cohen's *d*, Pearson's *r*), indicating how they were calculated |
| ☐ | ☒ | Clearly defined error bars<br>*State explicitly what error bars represent (e.g. SD, SE, CI)* |

*Our web collection on statistics for biologists may be useful.*

## Software and code

Policy information about availability of computer code

| | |
|---|---|
| Data collection | N/A |
| Data analysis | Flow cytometry data were analyzed using FloJo version 10.0.7r2. Images were prepared with Illustrator. Statistical analysis was performed with Graphpad Prism version 6. |

For manuscripts utilizing custom algorithms or software that are central to the research but not yet described in published literature, software must be made available to editors/reviewers upon request. We strongly encourage code deposition in a community repository (e.g. GitHub). See the Nature Research guidelines for submitting code & software for further information.

## Data

Policy information about availability of data

All manuscripts must include a data availability statement. This statement should provide the following information, where applicable:

- Accession codes, unique identifiers, or web links for publicly available datasets
- A list of figures that have associated raw data
- A description of any restrictions on data availability

The datasets generated during and/or analyzed during the current study are available from the corresponding author on reasonable request.

# Field-specific reporting

Please select the best fit for your research. If you are not sure, read the appropriate sections before making your selection.

☒ Life sciences ☐ Behavioural & social sciences ☐ Ecological, evolutionary & environmental sciences

For a reference copy of the document with all sections, see nature.com/authors/policies/ReportingSummary-flat.pdf

# Life sciences study design

All studies must disclose on these points even when the disclosure is negative.

| | |
|---|---|
| Sample size | The number of samples was the minimum number required to obtain scientifically valid results. |
| Data exclusions | No data were excluded from the analyses. |
| Replication | All attempts at replication in the stated conditions were successful |
| Randomization | Allocation of animals to experimental groups was random. |
| Blinding | There was no blinding. |

# Reporting for specific materials, systems and methods

## Materials & experimental systems

| n/a | Involved in the study |
|---|---|
| ☒ | ☐ Unique biological materials |
| ☐ | ☒ Antibodies |
| ☐ | ☒ Eukaryotic cell lines |
| ☒ | ☐ Palaeontology |
| ☐ | ☒ Animals and other organisms |
| ☒ | ☐ Human research participants |

## Methods

| n/a | Involved in the study |
|---|---|
| ☒ | ☐ ChIP-seq |
| ☐ | ☒ Flow cytometry |
| ☒ | ☐ MRI-based neuroimaging |

## Antibodies

| | |
|---|---|
| Antibodies used | rabbit anti-MARV GP (IBT # 0303-007); rabbit anti-MARV VP40 (IBT cat # 0303-001); goat anti-human antibody conjugated to 6 nm gold particles (Aurion, Netherlands); HRP-conjugated goat anti-human IgG (KPL Gaithersburg, MD, # 474-1002); Donkey anti-guinea pig-IgG Cy5 conjugated (Jackson ImmunoResearch, # 706-175-148); human anti-MARV antibodies MR78, MR186, MR235 are kind gifts of Dr. Crowe, Vanderbilt University; anti-human CD107a (Clone H4A3, BD Biosciences #555802), anti-human IFNγ (Clone B27, BD Biosciences #554702), anti-human MIP-1β (Clone D21-1351, BD Biosciences #550078); anti-human CD66b (Clone G10F5; Biolegend #305112); anti-human CD3 (Clone UCHT1; BD Biosciences #557943); anti-human CD14 (Clone MP9; BD Biosciences #557831) |
| Validation | See manufacturer's website for validation. |

## Eukaryotic cell lines

Policy information about cell lines

| | |
|---|---|
| Cell line source(s) | Vero E6, DF-1 and HEK293 cell lines were obtained from the ATCC. |
| Authentication | N/A |
| Mycoplasma contamination | Yes. Vero E6 cells were tested for mycoplasma contaminnation by PCR. |
| Commonly misidentified lines (See ICLAC register) | N/A |

## Animals and other organisms

Policy information about studies involving animals; ARRIVE guidelines recommended for reporting animal research

| | |
|---|---|
| Laboratory animals | 9 week-old female Dunkin-Hartley guinea pigs |
| Wild animals | N/A |
| Field-collected samples | N/A |

## Flow Cytometry

### Plots

Confirm that:

☒ The axis labels state the marker and fluorochrome used (e.g. CD4-FITC).

☒ The axis scales are clearly visible. Include numbers along axes only for bottom left plot of group (a 'group' is an analysis of identical markers).

☒ All plots are contour plots with outliers or pseudocolor plots.

☒ A numerical value for number of cells or percentage (with statistics) is provided.

### Methodology

Sample preparation

Isolation of natural killer cells: Primary human NK cells were enriched from a buffy coat by negative selection with RosetteSep Human NK cell Enrichment cocktail (StemCell Cat #15065) according to manufacturer's instructions. NK cells were isolated using density centrifugation. NK cells were resuspended to a final concentration of 1.5 x 106 cells/ml in complete growth medium supplemented with 1ng/ml of recombinant human IL-15 (StemCell Cat #78031.1). Cells were incubated at 37ºC 5% CO2 overnight until ready for use in the assay. Following the assay, cells were stained with fluorophore-conjugated antibodies for 15 minutes at room temperature prior to fixation, permeabilization, and intracellular staining fluorophore-conjugated antibodies. Fixed samples were analyzed by flow cytometry within 24 hours of fixation.

Isolation of neutrophils: Peripheral whole blood from human donors was collected into an acid citrate dextrose (ACD) Vacutainer (BD). Ammonium chloride potassium (ACK) lysis buffer was used to lyse red blood cells. White blood cells were counted and resuspended to a final concentration of 5 x 105 cells/ml in complete growth medium for immediate use in the phagocytosis assay. The white blood cells were stained with fluorophore-conjugated antibodies for 15 minutes at room temperature prior to fixation with 4% paraformaldehyde. Fixed samples were analyzed by flow cytometry within 24 hours of fixation.

THP-1 cells were fixed with 4% paraformaldehyde, and fixed samples were analyzed by flow cytometry within 24 hours of fixation.

| | |
|---|---|
| Instrument | BD LSR II |
| Software | FlowJo v10 |
| Cell population abundance | A minimum of 10,000 events were captured for each sample. |
| Gating strategy | All cell populations were first gated on FSC-A and SSC-A. To gate on neutrophils, SSC-A high populations were selected, followed by gating on CD3negative, CD14negative, and CD66bpositive cells. NK cells were defined at CD3 negative, CD56 positive cells. As no additional markers were used for THP-1 cells, cells were gated by FSC-A and SSC-A only. |

☒ Tick this box to confirm that a figure exemplifying the gating strategy is provided in the Supplementary Information.
